# A Predictive Model for Cancer-Associated Thrombosis in Japanese Cancer Patients: Findings from the J-Khorana Registry

**DOI:** 10.1055/a-2207-7715

**Published:** 2024-01-08

**Authors:** Masaaki Shoji, Yugo Yamashita, Masanobu Ishii, Hitoki Inoue, Hiroshi Kato, Shin Fujita, Kazuhiro Matsui, Kazuko Tajiri, Mizuo Nameki, Nao Muraoka, Akiko Nonaka, Hiroshi Sugino, Mihoko Kono, Toru Oka, Daisuke Sueta, Issei Komuro, Kenichi Tsujita, on behalf of J-Khorana Registry Investigators

**Affiliations:** 1 Department of General Internal Medicine, National Cancer Center Hospital, Tokyo, Japan; 2 Department of Cardiovascular Medicine, Kyoto University Graduate School of Medicine, Kyoto, Japan; 3 Department of Cardiovascular Medicine, Kumamoto University School of Medicine, Kumamoto, Japan; 4 Department of Cardiology, National Hospital Organization (NHO) Hokkaido Cancer Center, Hokkaido, Japan; 5 Division of Onco-Cardiology, Miyagi Cancer Center, Miyagi, Japan; 6 Department of Surgery, Tochigi Cancer Center, Tochigi, Japan; 7 Department of Internal Medicine, Onco-Cardiology Unit, Saitama Cancer Center, Saitama, Japan; 8 Department of Cardiology, National Cancer Center Hospital East, Chiba, Japan; 9 Division of Cardiology, Chiba Cancer Center, Chiba, Japan; 10 Division of Cardiology, Shizuoka Cancer Center, Shizuoka, Japan.; 11 Division of Onco-Cardiology, Hyogo Cancer Center, Hyogo, Japan; 12 Division of Cardiology, NHO Kure Medical Center and Chugoku Cancer Center, Hiroshima, Japan; 13 Department of Onco-Cardiology, NHO Kyushu Cancer Center, Fukuoka, Japan; 14 Department of Frontier Cardiovascular Science, International University of Health and Welfare, Tokyo, Japan; 15 Department of Cardiovascular Medicine, Graduate School of Medicine, The University of Tokyo, Tokyo, Japan

**Keywords:** venous thromboembolism, cancer, predictive model

## Abstract

**Background** Although the close relationship between cancer and venous thromboembolism (VTE) has been identified, risk stratification for VTE in Japanese patients with cancer remains unclear.

**Objectives** This study aimed to validate the Khorana VTE risk assessment score (KRS) for VTE diagnosis and establish an optimal predictive model for VTE in Japanese patients with cancer.

**Methods** A total of 7,955 Japanese patients with cancer were subdivided into low- (0), intermediate- (1–2), and high-score (3) groups according to the KRS. Using 37 explanatory variables, a total of 2,833 patients with cancer were divided into derivation and validation cohorts (5:5). A risk model for Japanese participants was developed using the derivation cohort data.

**Results** The prevalence of VTE in low-, intermediate-, and high-score patients was 1.2, 2.5, and 4.3%, respectively. Logistic regression analysis demonstrated that cancer stage (III–IV) and KRS ≥ 2 were independent and significant predictors of VTE onset. The risk model for VTE assigned 1 point to body mass index ≥25 kg/m ^2^ and 2 points each to the prevalence of osteochondral cancer and D-dimer level ≥1.47 µg/mL. The areas under the curve of the risk model were 0.763 and 0.656 in the derivation and validation cohorts, respectively.

**Conclusion** The KRS was useful in Japanese patients, and our new predictive model may be helpful for the diagnosis of VTE in Japanese patients with cancer.

## Introduction

**Graphical Abstract:**
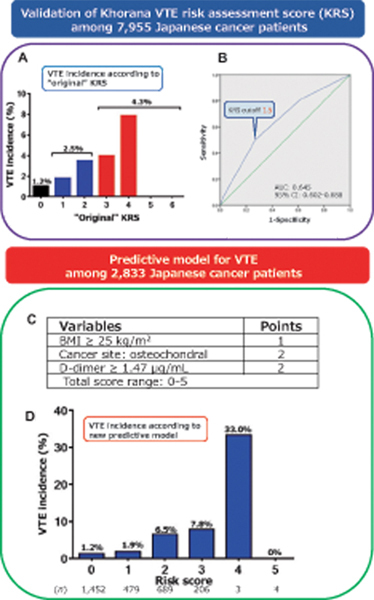
( **A** ) VTE incidence in the enrolled patients according to the original KRS. ( **B** ) ROC curve of the KRS for the prediction of VTE onset. ( **C** ) VTE predictors in the derivation cohort according to the multivariate logistic regression analysis. ( **D** ) VTE incidence according to the new predictive model. BMI, body mass index; KRS, Khorana venous thromboembolism risk assessment score; ROC, receiver operating characteristic; VTE, venous thromboembolism.

The susceptibility to thrombosis of patients with cancer has been well-known since Jean-Baptiste Bouillaud described three patients with cancer and deep vein thrombosis in 1823. The concept of cancer-associated venous thromboembolism (VTE) has been recently updated. 1 In a meta-analysis, the 1-year risk of developing VTE in patients with cancer was estimated to be 43 per 1,000. 2 Moreover, a subanalysis of the Computerized Registry of Patients with Venous Thromboembolism (RIETE) registry reported that patients with cancer had a significantly higher mortality rate due to pulmonary embolism. 3

In 2008, Khorana et al proposed a prechemotherapy VTE risk assessment score for patients with cancer, the Khorana VTE risk assessment score (KRS; Supplementary Table S1 , available in the online version>). 4 A recent review revealed that a KRS score of ≥2 was a significant thrombotic risk factor that should be considered for prophylactic VTE treatment. 5 In the latest guidelines, thromboembolic and bleeding risk reassessments are strongly recommended for patients with cancer. 6 Since the proposal of KRS, various scoring methods, such as the Vienna score, 7 have emerged. 8 9 10 11 12 13 Recently, these concepts were comprehensively reviewed. 14 The original 15 and modified 16 Ottawa scores are useful tools for stratifying the risk of recurrence of cancer-associated thrombosis (CAT). However, most of these studies focused on Western patients, and the different genetic background and physique of Asian patients will lead to different risks of developing VTE. 17 18 Whether such scoring systems can be applied to East Asian patients is questionable, and it is essential to establish suitable scoring methods for East Asian patients. Although previous studies have demonstrated the prediction of VTE in East Asian individuals, 19 20 21 22 23 these studies used relatively small cohorts and included only specific cancer sites.

Recently, we validated the KRS for diagnosing VTE in Japanese patients with cancer. 24 However, such report was performed at a single center. Hence, this study attempted to verify the validity of the KRS in diagnosing VTE using a multicenter registry and then establish an optimal predictive model for Japanese patients.

## Methods

This retrospective, multicenter, observational study explored the clinical outcomes of patients with cancer. This study was conducted jointly by the Japanese Onco-Cardiology Society.

### Ethical Approval

All the procedures were conducted in accordance with the Declaration of Helsinki and its amendments. The study protocol was approved by the Institutional Review Board of Kumamoto University (approval number, Rinri 2308) and those of all participating institutions. This study was registered in the University Hospital Medical Information Network Clinical Trials Registry (UMIN000050391). The opt-out procedure was demonstrated at each institution to all patients.

### Study Participants

The J-Khorana Registry is a multicenter, retrospective, observational study conducted to develop a predictive model for CAT in Japanese patients with cancer. This registry included 9,965 patients newly diagnosed with cancer throughout Japan (11 cancer centers; details are described in Supplementary Appendix 1 , available in the online version>) between April 2019 and June 2019. All data were collected and aggregated by a trained research team from the Division of Cardiovascular Disease at Kumamoto University.

### Study Outline

**Fig. 1:**
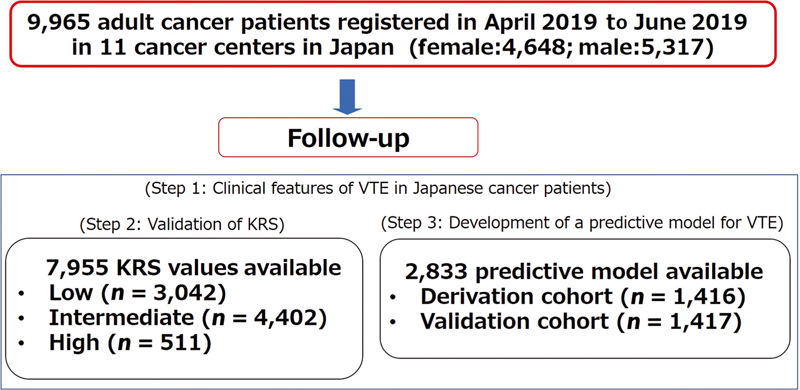
Study flowchart. KRS, Khorana risk assessment score; VTE, venous thromboembolism.

The outline of this study is shown in Fig. 1 . This study consisted of the following three steps:

Step 1: Clarification of clinical features of VTE in Japanese patients with cancer
Step 2: Validation of the KRS
Step 3: Development of a predictive model for VTE

A detailed explanation of each step is included in the “Results” section.

### Calculation of the Khorana Venous Thromboembolism Risk Assessment Score

The KRS was calculated as described previously ( Supplementary Table S1 , available in the online version>). 4 In brief, the KRS comprised the following five clinical items: tumor site (stomach and pancreatic cancers, classified as “very high risk”; lung, lymphoma, gynecological, bladder, or testicular cancer, classified as “high risk”), prechemotherapy platelet count of ≥350 × 10 ^9^ /L, prechemotherapy hemoglobin concentration of <10 g/dL, and/or the use of erythropoiesis-stimulating agents, prechemotherapy leukocyte count of >11 × 10 ^9^ /L, and a body mass index (BMI) value of >35 kg/m ^2^ . According to the World Health Organization Asian classification defined by expert consultation 25 and based on the typical body shape of Asian populations, a BMI of ≥25 kg/m ^2^ is defined as obesity. This methodology has been widely used in clinical studies. 22 24 26

### Clinical Parameters

Baseline demographic data at enrollment were collected. Estimated glomerular filtration rate (eGFR) was calculated using the Japanese Society of Nephrology formula. 27 Chronic kidney disease was defined as an eGFR of ≤60 mL/min/1.73 m ^2^ .

### Venous Thromboembolism Diagnosis

VTE was diagnosed by well-trained cardiologists. VTE was defined according to appropriate diagnostic criteria 28 and confirmed using enhanced computed tomography or lower extremity ultrasound.

### Follow-up and Endpoints

After enrollment, the patients were followed up at the outpatient clinic for 1 year or until an endpoint was reached. The primary endpoint was the onset of VTE. The endpoints were ascertained from a review of the medical records and confirmed by direct contact with the patients, their families, and their physicians, or an annual telephone interview conducted with each patient.

### Statistical Analysis

Continuous variables are expressed as median values with interquartile ranges. Categorical data were presented as numbers or percentages. The data were analyzed with the χ ^2^ test for categorical variables and the Kruskal–Wallis test followed by post hoc Dunn's multiple comparison test for continuous variables among the comparison groups, as appropriate. We used the Kaplan–Meier method to estimate the secondary endpoint probabilities, and the log-rank test to compare the distributions of survival times among the groups. A logistic regression model was used to calculate odds ratios. Receiver operating characteristic (ROC) curves were generated and 95% confidence intervals (CIs) were calculated to assess the predictive ability of the KRS, and the Youden index was used to determine the optimal cutoff point. 29 The Youden index is defined as the maximum vertical distance between the ROC curve and the diagonal or chance line and is calculated as follows: Youden index = maximum (sensitivity + specificity − 1). Using this measure, the cutoff point on the ROC curve that corresponds to the Youden index, that is, at which (sensitivity + specificity − 1) is maximized, is selected as the optimal cutoff point. An intuitive interpretation of the Youden index is that it corresponds to the point on the curve that is farthest from a random classification. 30

Based on our previous report 24 and considering their clinical relevance, we selected 37 potential variables for VTE, including 28 clinical variables and 9 laboratory variables (details are described in Supplementary Appendix 2 , 1 available in the online version>). A total of 2,833 cases with no missing values for 37 explanatory variables were extracted from the cohort of 9,965 patients and randomly assigned to derivation and validation cohorts in a 5:5 ratio, stratified by the outcome. A risk model was developed from the derivation cohort data. A univariate logistic regression model was developed to assess the association between the 37 explanatory variables and the presence of VTE events in the derivation cohort. We developed a risk model using the results of the multivariable stepwise logistic regression models, in which we divided the respective β coefficient by the smallest β coefficient and rounded it to the nearest variable value. The accuracy of the predictive model in the derivation and validation cohorts was evaluated using an ROC curve analysis.

Statistical significance was set at *p* -value <0.05. All statistical analyses were performed using SPSS version 26 (IBM Corp., Armonk, NY).

## Results

### Patient Characteristics (Step 1)

The three most common malignant diseases in the female patients were breast, gynecological, and lung cancers ( Supplementary Fig. S1A , available in the online version), and those in male patients were lung, large intestine, and prostate cancers ( Supplementary Fig. S1B , available in the online version). Of the 9,965 enrolled patients, 196 (1.97%) experienced VTE onset during the observation period. Table 1 shows the baseline characteristics of patients in the no-VTE ( *n* = 9,769) and VTE ( *n* = 196) groups. Among the clinical features examined, male sex, cancer stage (0–II), blood hemoglobin concentration, and serum total protein (TP) concentration in the no-VTE group were significantly higher than those in the VTE group, whereas the C-reactive protein (CRP) level, plasma D-dimer level, and KRS in the no-VTE group were significantly lower than those in the VTE group.

**Table 1:** Baseline characteristics of enrolled cancer patients

	All patients *n* = 9,965	No-VTE *n* = 9,769	VTE *n* = 196	*p* -Value
Age, years	68 (57–75)	68 (57–75)	68 (59–76)	0.669
Male (%)	5,317 (53.4)	5,233 (53.6)	84 (42.9)	0.003
BMI, kg/m ^2^	22.3 (19.9–24.8)	22.3 (19.9–24.8)	22.5 (19.5–25.8)	0.988
BSA, m ^2^	1.59 (1.47–1.72)	1.59 (1.47–1.72)	1.61 (1.43–1.73)	0.992
Cancer stage				
0 to II (%)	4,566 (45.8)	4,508 (46.1)	58 (29.6)	<0.01
III to IV (%)	2,783 (27.9)	2,682 (27.5)	101 (51.5)	<0.01
CKD (%)	1,534/5,827 (26.3)	1,492/5,686 (26.2)	42/141 (29.8)	0.345
WBC, /μL	5,800 (4,510–7,300)	5,760 (4,500–7,300)	6,400 (5,205–8,400)	<0.01
RBC, /μL	427 (385–464)	428 (386–464)	406 (360–458)	0.325
Hemoglobin, g/dL	13.2 (12.0–14.2)	13.2 (12.0–14.3)	12.3 (10.3–13.7)	<0.01
Platelet, 10 ^3^ /μL	27.5 (21.1–128.0)	27.5 (21.1–131.5)	28.1 (20.4–47.8)	0.993
TP, g/L	7.1 (6.7–7.4)	7.1 (6.7–7.4)	6.9 (6.4–7.3)	<0.01
Albumin, g/dL	4.1 (3.7–4.4)	4.2 (3.8–4.4)	3.9 (3.1–4.2)	0.244
AST, U/L	21 (17–27)	21 (17–27)	22 (18–31)	0.227
ALT, U/L	17 (13–24)	17 (13–24)	18 (12–28)	0.599
T-Bil, mg/dL	0.60 (0.5–0.8)	0.60 (0.5–0.8)	0.6 (0.4–0.7)	0.874
BUN, g/dL	14.6 (11.9–18.0)	14.6 (11.9–18.0)	15.0 (11.5–19.0)	0.435
Cr, mg/dL	0.75 (0.62–0.91)	0.75 (0.62–0.91)	0.72 (0.60–0.88)	0.990
eGFR, mL/min/1.73 m ^2^	70.4 (59.3–82.7)	70.5 (59.4–82.5)	70.3 (56.3–89.1)	0.353
UA, mg/dL	5.1 (4.2–6.2)	5.1 (4.2–6.2)	4.9 (4.1–6.1)	0.536
T-Chol, mg/dL	197 (171–224)	197 (171–224)	193 (180–215)	0.788
LDL, mg/dL	112 (86–141)	112 (86–142)	108 (94–129)	0.677
HDL, mg/dL	56 (43–72)	56 (43–73)	55 (37–68)	0.064
TG, mg/dL	91 (57–137)	91 (57–137)	104 (75–153)	0.109
CRP, mg/dL	0.17 (0.05–1.07)	0.16 (0.05–1.01)	0.64 (0.11–4.68)	<0.01
HbA1c, %	6.0 (5.6–6.7)	6.0 (5.6–6.7)	5.9 (5.6–6.5)	0.427
D-dimer, µg/mL	0.9 (0.5–2.1)	0.8 (0.5–2.0)	2.3 (0.9–7.3)	<0.01
KRS, points	0.97 ± 0.94	0.96 ± 0.94	1.47 ± 1.01	<0.01

Abbreviations: Albumin, serum albumin concentration; ALT, alanine aminotransferase concentration; AST, aspartate aminotransferase concentration; BMI, body mass index; BSA, body surface area; BUN, blood urea nitrogen; CKD, chronic kidney disease; Cr, serum creatinine concentration; CRP, plasma C-reactive protein concentration; eGFR, estimated glomerular filtration rate; HbA1c, hemoglobin A1c level; HDL, serum high-density lipoprotein cholesterol concentration; Hemoglobin, blood hemoglobin level; KRS, Khorana venous thromboembolism risk assessment score; LDL, serum low-density lipoprotein cholesterol concentration; Platelet, blood platelet count; RBC, red blood cell count; T-Bil, total bilirubin concentration; T-Chol, serum total cholesterol concentration; TG, serum triglyceride concentration; TP, serum total protein concentration; UA, serum uric acid concentration; VTE, venous thromboembolism; WBC, white blood cell count.

Data are presented as median (interquartile range) or number (percentage).

The detailed ROC curves for the complete blood count profile (platelet count, hemoglobin level, and leukocyte count), BMI, and hemostatic measures (CRP and D-dimer) are shown in Supplementary Fig. S2 (available in the online version).

### Venous Thromboembolism Prevalence in Patients with Cancer (Step 2)

A total of 7,955 cases with no missing values for 5 variables (the original Khorana score components described in Supplementary Table S1 , available in the online version>) were extracted from the cohort of 9,965 patients.

**Fig. 2:**
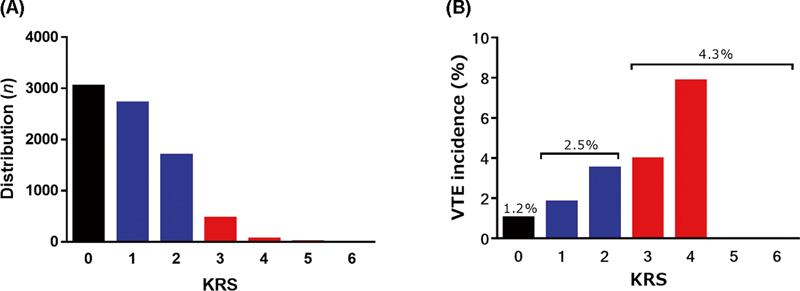
Patients distribution (A) and VTE incidence (B) in the enrolled patients according to the KRS. KRS, Khorana venous thromboembolism risk assessment score; VTE, venous thromboembolism.

Fig. 2A shows the KRS distribution of the enrolled patients. The incidence of VTE was 1.2, 2.5, and 4.3% in the low, intermediate, and high KRS groups, respectively ( Fig. 2B ).

### Receiver Operating Characteristic Analysis of the KRS for Venous Thromboembolism Onset and Predictors of Venous Thromboembolism Onset (Step 2)

**Fig. 3:**
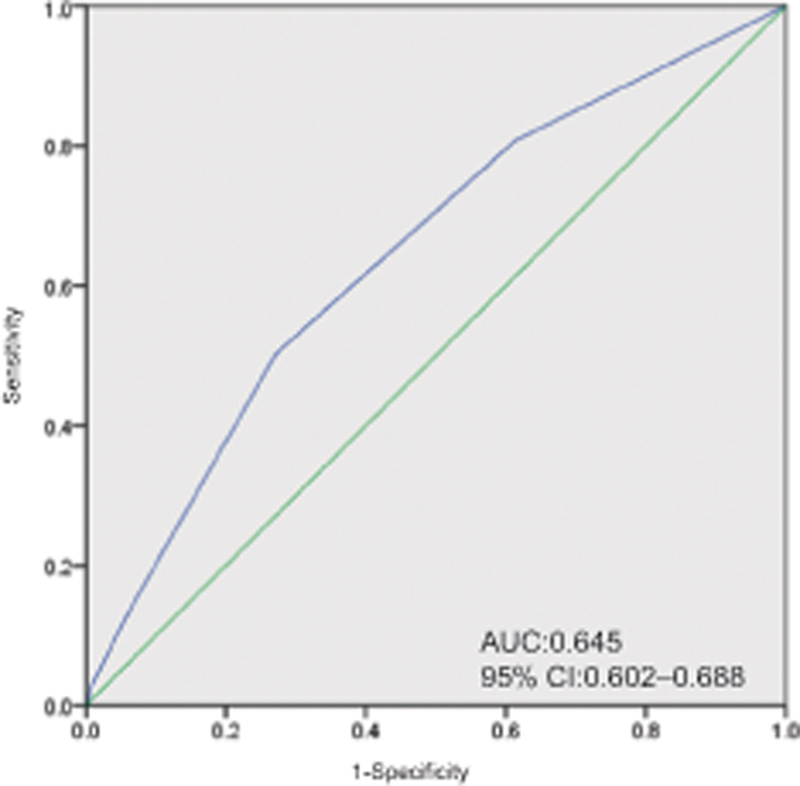
ROC curve of the KRS for VTE onset prediction. AUC, area under the curve; CI, confidence interval; KRS, Khorana venous thromboembolism risk assessment score; ROC, receiver operating characteristic; VTE, venous thromboembolism.

An ROC curve was constructed to assess the ability of the KRS to diagnose VTE onset ( Fig. 3 ). The area under the curve (AUC) of the KRS for the detection of VTE onset was 0.645 (95% CI = 0.602–0.688). For a KRS cutoff of 1.5, the sensitivity and specificity were 50.3 and 72.8%, respectively.

In the univariable logistic regression analyses of VTE onset, cancer stage (0–II), cancer stage (III–IV), TP, serum albumin concentration, CRP, plasma D-dimer level, KRS, cancer site (1 point), cancer site (0 point), platelet count > 350 × 10 ^9^ /L, blood hemoglobin < 10.0, white blood cell count > 11 × 10 ^9^ /L, BMI ≥ 25, KRS category, and KRS ≥ 2 were revealed to be potential significant determinants of VTE onset in patients with cancer ( Table 2 ). In the multivariable logistic regression analysis of VTE onset, cancer stage (III–IV) and KRS ≥ 2 were independent and significant predictors of VTE onset ( Table 2 ).

**Table 2:** Logistic regression for prediction of venous thromboembolism

Variable	Univariable regression			Multivariable regression stepwise backward		
	OR	95% CI	*p*	OR	95% CI	*p*
Age, years	0.996	0.985–1.008	0.539	–	–	–
Male sex	0.734	0.537–1.004	0.053	–	–	–
BMI, kg/m ^2^	1.000	0.985–1.017	0.957	–	–	–
BSA, m ^2^	1.004	0.430–2.344	0.992	–	–	–
Cancer stage						
0 to II	0.438	0.309–0.621	<0.01	–	Not selected	–
III to IV	2.482	1.815–3.393	<0.01	2.316	1.691–3.173	<0.01
Chronic kidney disease	1.079	0.710–1.639	0.721	–	–	–
TP, g/L	0.598	0.470–0.759	<0.01	–	Not selected	–
Alb, g/L	0.834	0.727–0.958	<0.01	–	Not selected	–
eGFR, mL/min/1.73 m ^2^	1.006	0.997–1.014	0.213	–	–	–
AST, U/L	1.001	0.997–1.005	0.546	–	–	–
ALT, U/L	1.000	0.995–1.004	0.853	–	–	–
T-Bil, mg/dL	0.979	0.854–1.123	0.765	–	–	–
UA, mg/dL	0.967	0.869–1.075	0.532	–	–	–
LDL, mg/dL	0.999	0.992–1.005	0.678	–	–	–
HDL, mg/dL	0.986	0.972–1.001	0.062	–	–	–
TG, mg/dL	1.001	1.00–1.003	0.095	–	–	–
HbA1c, %	0.997	0.991–1.004	0.416	–	–	–
CRP, mg/dL	1.040	1.013–1.068	0.004	–	Not selected	–
D-dimer, µg/dL	1.035	1.021–1.049	<0.01	–	Not selected	–
KRS	1.661	1.433–1.925	<0.01	–	Not selected	–
Cancer site						
2 points	1.262	0.845–1.886	0.256	–	Not selected	–
1 point	1.890	1.378–2.593	<0.01	–	Not selected	–
0 point	0.487	0.354–0.672	<0.01	–	Not selected	–
Platelet count ≥350 × 10 ^9^ /L	3.843	2.091–7.062	<0.01	–	Not selected	–
Hemoglobin level <10.0 g/dL	3.034	2.021–4.554	<0.01	–	Not selected	–
Leukocyte count >11 × 10 ^9^ /L	2.463	1.492–4.064	<0.01	–	Not selected	–
BMI ≥ 25 kg/m ^2^	1.463	1.039–2.059	0.029	–	Not selected	–
KRS categories						
Low	0.379	0.255–0.562	<0.01	–	Not selected	–
Intermediate	1.661	1.192–2.314	0.03	–	Not selected	–
High	2.364	1.494–3.741	<0.01	–	Not selected	–
KRS ≥ 2	2.712	1.983–3.708	<0.01	2.545	1.858–3.4886	<0.01

Abbreviations as shown in Table 1 ; CI, confidence interval; OR, odds ratio.

### Development of a Risk Model for Venous Thromboembolism in Japanese Patients with Cancer (Step 3)

The differences in baseline characteristics between the cohorts, whether included in the new prediction model or not, are reported in Supplementary Table S2 (available in the online version).

**Fig. 4:**
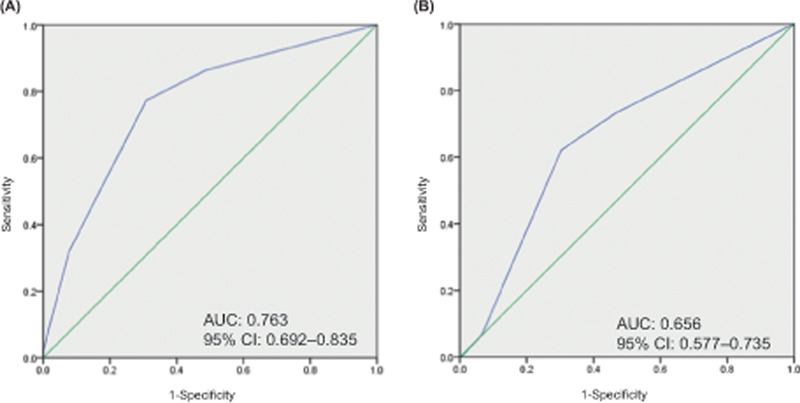
ROC curves of the risk model for VTE onset prediction. The ROC curves of the ( **A** ) deviation and ( **B** ) validation cohorts are shown. AUC, area under the curve; CI, confidence interval; KRS, Khorana venous thromboembolism risk assessment score; ROC, receiver operating characteristic; VTE, venous thromboembolism.

In the univariate and multivariate logistic regression analyses of VTE onset, BMI ≥ 25 kg/m ^2^ , plasma D-dimer level, and prevalence of osteochondral cancer were independent and significant positive predictors of VTE onset ( Table 3 ). Based on the Youden index described in the “Methods,” the cutoff value for the plasma D-dimer level was 1.465 µg/mL( Supplementary Fig. S3 , available in the online version). According to the results of the univariate and multivariate logistic regression models, the risk model for VTE assigned 1 point to BMI ≥ 25 kg/m ^2^ and 2 points each to the presence of osteochondral cancer and D-dimer values ≥1.47 µg/mL. The AUCs of the prediction model for VTE were 0.763 and 0.656 in the derivation ( Fig. 4A ) and validation ( Fig. 4B ) cohorts, respectively.

**Table 3:** Predictors of venous thromboembolism in the derivation cohort by multivariate logistic regression analysis

Variables	OR	95% CI	*p* -value	Points
BMI ≥ 25 kg/m ^2^	2.304	1.203–4.412	0.012	1
Cancer site: osteochondral	7.900	1.564–39.91	0.012	2
D-dimer ≥ 1.47 µg/mL	8.165	3.950–16.87	<0.001	2

Abbreviations: BMI, body mass index; CI, confidence interval; OR, odds ratio.

Racial differences in the level of BMI 25 and D-dimer 31 have been reported, which may partly explain the low rate of VTE in the East Asian population examined here. The number of patients within each category of BMI and D-dimer levels, according to J-Khorana scores, is showcased in Table 4 .

**Table 4:** The proportion of patients in the levels of body mass index and D-dimer according to J-Khorana score

Variables	Overall	0	1	2	3	4	5	6
Number of patients	2,833	1,452	479	689	206	3	4	0
VTE (%)	89 (3.1)	18 (1.2)	9 (1.9)	45 (6.5)	16 (7.8)	1 (33.4)	0 (0)	N/A
BMI, kg/m ^2^	22.4 (19.9–24.9)	21.4 (19.5–23.0)	27.1 (26.0–29.0)	21.1 (18.8–23.0)	27.3 (26.0–29.8)	22.8 (21.0–23.6)	27.1 (25.4-29.7)	N/A
D-dimer, µg/mL	0.8 (0.5–2.0)	0.5 (0.4–0.9)	0.5 (0.3–0.9)	3.5 (2.1–6.3)	3.4 (2.0–6.0)	2.5 (2.1–5.3)	4.8 (1.8-7.6)	N/A

Abbreviations: BMI, body mass index; CI, confidence interval; OR, odds ratio; VTE, venous thromboembolism.

Data are presented as median (interquartile range) or number (percentage).

## Discussion

This study validates KRS for Japanese patients (AUC = 0.645) and the higher the KRS score, the higher the VTE incidence in Japanese patients with cancer (Step 2). However, this AUC value was not so high, and it was necessary to establish a Japanese-specific risk model. Hence, our new risk model using BMI, cancer sites, and plasma D-dimer levels was validated in Japanese participants (Step 3).

Several clinical studies have demonstrated a close relationship between cancer and the development of thrombosis, known as CAT. 32 CAT includes cancer-associated VTE, 14 arterial thromboembolism, 33 and cancer-associated nonbacterial thrombotic endocarditis. 34 VTE is often asymptomatic, and in patients with cancer, is often diagnosed incidentally; for example, when staging the cancer, searching for metastatic lesions, and assessing therapeutic effects. Galanaud et al revealed that patients with cancer-related isolated distal VTE have a much poorer prognosis than those with isolated distal VTE without cancer, and the prognoses of patients with cancer-related isolated distal VTE or cancer-related proximal VTE patients are similar. 35 Therefore, early VTE diagnosis is of extreme importance, especially in patients with cancer.

In the present study, cancer stage (III–IV) and KRS ≥ 2 were independent and significant predictors of VTE onset ( Table 2 ). Advanced cancer is presumed to be a risk factor for VTE because of its long history and has been reported as a common cancer-related risk factor for VTE in patients with cancer. 36 Moreover, it is reasonable to assume that an increased KRS is a risk factor for VTE.

The KRS has been extensively used for the risk assessment of CAT onset. The KRS scoring system is based on data from Western patients that can be easily and accurately calculated, including BMI among its factors. As Japanese patients have a relatively small physique, 22 the direct use of the KRS in these patients might not be feasible. Obesity has long been known as a risk factor for VTE 36 and has been used in several VTE risk assessment scores. 37 38 In the present study, we clarified that BMI is a predictor of CAT onset in Japanese patients with cancer.

Elevated D-dimer levels have been associated with the development of VTE in patients with cancer. 19 In case of positive D-dimer levels, additional tests, such as lower extremity vein echocardiography, are considered; however, the cutoff D-dimer value for positivity is not clear. The Vienna score, which is an improved version of the KRS, uses D-dimer levels; the optimal D-dimer level in the Vienna score is 1.44 g/L. Hamamoto et al established a pretest probability score for detecting preoperative VTE that was not limited to patients with cancer, with a D-dimer level ≥1.5 µg/mL as a risk factor. 39 This value is consistent with the D-dimer threshold established in our study (1.47 µg/mL).

Cancer cells release microparticles containing large amounts of tissue factors and activate the coagulation system. Microparticles are particularly common in pancreatic and mucinous cancers, and cohort studies have shown that thrombosis is common in these cancer types. 40 41 However, in the present study, the prevalence of osteochondral cancer was a risk factor for CAT development. The reason for this difference remains unclear; nonetheless, it results from the different genetic backgrounds of Western and Japanese patients. However, as bone cancer has also been classified as “high risk,” 40 our results may be reasonable. Therefore, further pathophysiological and molecular physiological studies, including animal experiments, are required.

To the best of our knowledge, this is the first study to establish a predictive model for VTE in Japanese patients with cancer. Each component of the new predictive model is simple to obtain, and the calculation is not only easy in clinical practice but is also low cost, which indicates that the predictive model can be widely applied. However, the validity of this scoring system was demonstrated in the same cohort, if this scoring system could be validated in external validation cohorts, it could also serve as a useful indicator for oncologists and cardiologists in clinical settings. Although the new predictive model is expected to have a high clinical value, large-scale clinical studies, including external validation steps, are needed to confirm its true value.

## Study Limitation

The present study had some limitations. First, during the development of the prediction model, a large number of cases with no explanatory variables were excluded from the overall cohort. Second, the predictive model in the present study was validated in the same cohort; therefore, further validation in an external cohort is warranted. The prediction model obtained in the present study has not undergone external validation; thus, this model must be considered a candidate model until external validation is obtained. Furthermore, it is unclear which factors contribute to the development of VTE in patients with osteochondral cancer and the extent of their contribution. Therefore, further pathophysiological and molecular physiological studies, including animal experiments, are required. Additional detailed large-scale clinical studies are required to verify the utility of our predictive model. Finally, although whole blood viscosity (WBV) using total protein (TP) and hematocrit levels can be estimated, 42 it was not possible to estimate WBV values due to lacking data. Therefore, evaluating the predictive ability of VTE occurrence using WBV values was not possible in the present study.

## Conclusion

To the best of our knowledge, this study is the first to clearly provide an optimal predictive model of VTE for Japanese patients with cancer based on a multicenter registry, providing new insights into oncocardiology.
