# Supplementary material for: A Predictive Model for Cancer-Associated Thrombosis in Japanese Cancer Patients: Findings from the J-Khorana Registry
Source: TH Open. 2024 Jan 8;8(1):e9–e18. doi: 10.1055/a-2207-7715 (PMC10774015; doi:10.1055/a-2207-7715)
Supplement: Supplementary file 1 — Supplementary Material [file 10-1055-a-2207-7715-s23100042.pdf]

SupplementaryMaterials

Appendix 1

Hospitals participating in the J-Khorana Registry

- National Hospital Organization (NHO) Hokkaido Cancer Center, Sapporo, Japan
- Miyagi Cancer Center, Natori, Japan
- Tochigi Cancer Center, Tochigi, Japan
- Saitama Cancer Center, Saitama, Japan
- National Cancer Center Hospital East, Kashiwa, Japan
- Chiba Cancer Center, Chiba, Japan
- National Cancer Center Hospital, Tokyo, Japan
- Shizuoka Cancer Center, Sunto-gun, Shizuoka, Japan
- Hyogo Cancer Center, Akashi, Japan
- NHO Kure Medical Center and Chugoku Cancer Center, Kure, Japan
- NHO Kyushu Cancer Center, Fukuoka, Japan

Appendix 2

The 37 explanatory variables comprised 28 clinical variables (male sex, age, Body mass index, cancer stage [III–IV], stomach cancer, pancreas cancer, lung cancer, leukemia/malignant lymphoma, gynecology cancer, bladder cancer, testis cancer, orofacial cancer, ear/nose/throat cancer, esophagus cancer, endocrine cancer, breast cancer, small intestine cancer, liver cancer, bile duct cancer, large intestine cancer, peritoneum cancer, bone/cartilage cancer, kidney cancer, skin cancer, brain/central nervous system cancer, respiratory system cancer, prostate cancer and urinary tract cancer, and nine laboratory variables (WBC, Hemoglobin, Platelet, TP, T-Bil, Cr, BUN, CRP, D-dimer). Abbreviations as shown in ►**Table 1**.

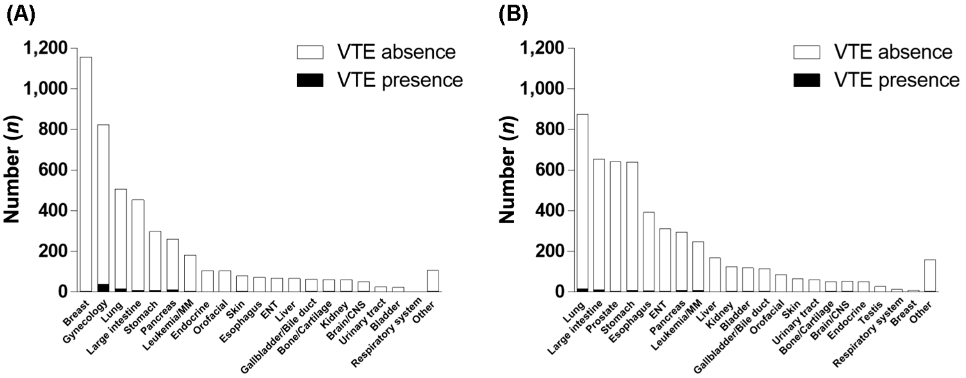

Supplementary Fig. S1 Distribution of malignant diseases of 9,965 adult patients awith cancer.(**A**) Females. (**B**) Males.CNS, central nervous system;ENT, ear/nose/throat; MM, malignant lymphoma; VTE, venous thromboembolism.

 © 2024. The Author(s).

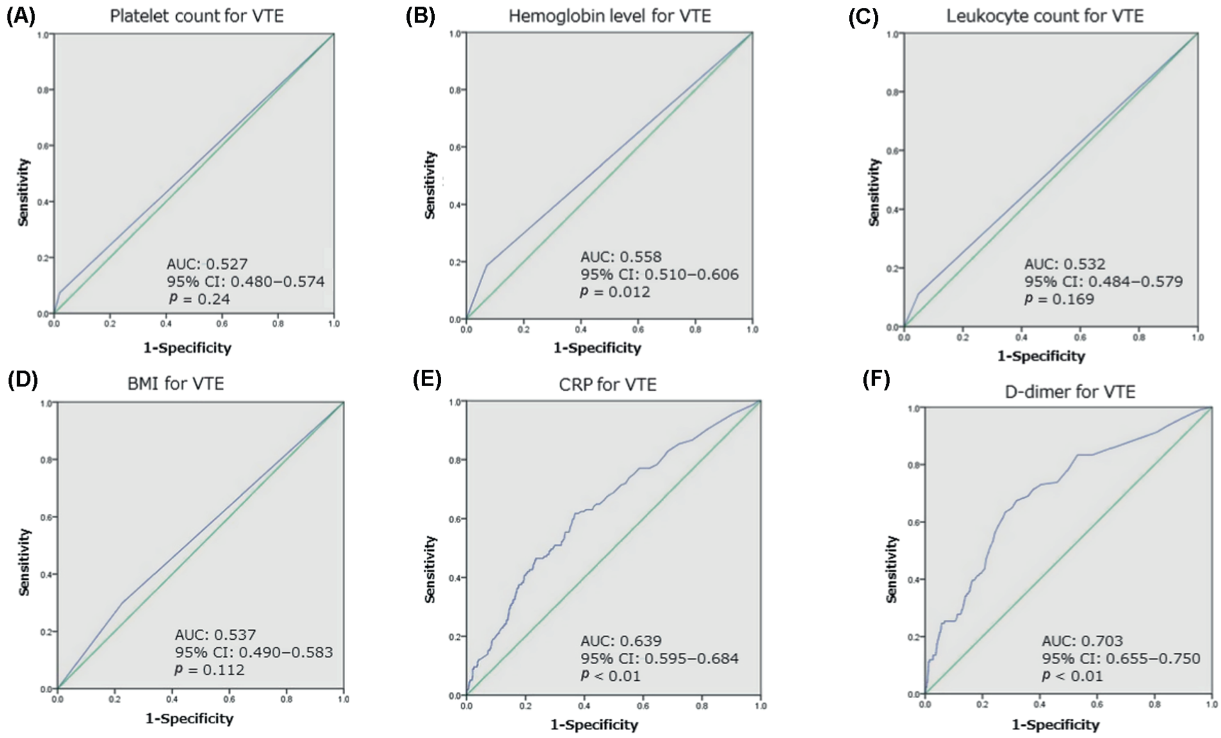

Supplementary Fig. S2 The detailed ROC curves for complete blood count profile (platelet count (A), hemoglobin level (B), and leukocyte count (C)), BMI (D), and hemostatic measures (CRP (E) and D-dimer (F); Step 1). AUC, area under the curve; BMI, body mass index; CI, confidence interval; CRP, C-reactive protein; ROC, receiver operating characteristic; VTE, venous thromboembolism.

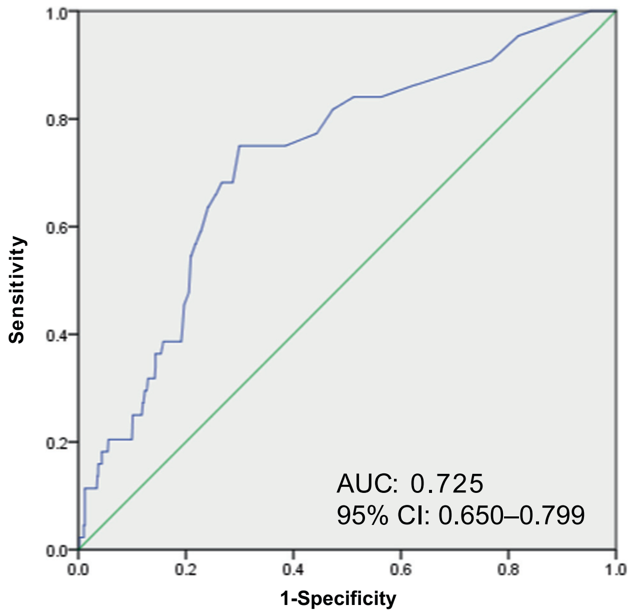

Supplementary Fig. S3 ROC curve of plasma D-dimer level for the prediction of VTE onset (Step 2).AUC, area under the curve; CI, confidence interval; ROC, receiver operating characteristic; VTE, venous thromboembolism.

 © 2024. The Author(s).

Supplementary Table S1 Khorana venous thromboembolism risk assessment score for prediction of venous thromboembolism in cancer patients

Variable	Points
**Site of cancer**	
Very high risk cancer (stomach, pancreas)	2
High risk cancer (lung, lymphoma, gynecological, bladder, or testicular)	1
**Prechemotherapy platelet count $\geq 350 \times 10^9$/L**	1
**Prechemotherapy hemoglobin level <10.0 g/dL or use of red cell growth factors**	1
**Prechemotherapy leukocyte count $>11 \times 10^9$/L**	1
**Body mass index $\geq$ 35 kg/m^2[a]**	1
**Traditional risk categories**	
High	≥3
Intermediate	1–2
Low	0

This table is adapted (and modified) from Khorana et al 2008[4].
[a]According to the World Health Organization Asian classification defined by expert consultation[25] based on the typical body shape of the Asian populations, a body mass index value of 25 kg/m^2 or more was defined as obesity.

Supplementary Table S2 Baseline characteristics of the cohorts based on their inclusion in the new prediction model

	All patients *n* = 9,965	Cohort excluded *n* = 7,132	Cohort included *n* = 2,833	*p*-value
Age, years	68 (57–75)	68 (56–75)	67 (57–75)	0.114
Male, n (%)	5,317 (53.4)	3,802 (53.3)	1,515 (53.5)	0.88
BMI, kg/m^2	22.3 (19.9–24.8)	22.3 (20.0–24.8)	22.4 (19.9–24.9)	0.283
KRS points, *n* (%)				
0	3,042 (30.5)	2,140 (30.0)	902 (31.8)	–
1	2,713 (27.2)	1,710 (24.0)	1,003 (35.4)	–
2	1,689 (16.9)	987 (13.8)	702 (24.8)	–
3	455 (4.6)	254 (3.6)	201 (7.1)	–
4	51 (0.5)	28 (0.4)	23 (0.8)	–
5	5 (<0.1)	3 (<0.1)	2 (0.1)	–
Unknown	2,010 (20.1)	2,010 (28.2)	0 (0)	–
WBC, /μL	5,800 (4,510–7,300)	5,610 (4,400–7,100)	6,100 (4,800–7,700)	<0.001
Hemoglobin, g/dL	13.2 (12.0–14.2)	13.2 (12.0–14.2)	13.2 (12.0–14.4)	0.035
Platelet, 10^3/μL	28 (21–128)	28 (21–177)	26 (21–36)	<0.001
TP, g/L	7.1 (6.7–7.4)	7.0 (6.7–7.4)	7.1 (6.8–7.4)	<0.001
T-Bil, mg/dL	0.60 (0.5–0.8)	0.60 (0.5–0.8)	0.6 (0.5–0.8)	0.614
BUN, g/dL	14.6 (11.9–18.0)	14.6 (11.9–18.0)	14.4 (11.9–18.0)	0.3
Cr, mg/dL	0.75 (0.62–0.91)	0.75 (0.63–0.91)	0.75 (0.62–0.90)	0.219
CRP, mg/dL	0.15 (0.05–0.82)	0.14 (0.05–0.75)	0.16 (0.05–0.92)	0.82
D-dimer, μg/mL	0.9 (0.5–2.1)	1.0 (0.5–2.8)	0.8 (0.5–2.0)	<0.001

Abbreviations as shown in ►**Tables 1** and **2**.

 © 2024. The Author(s).
